# Pathogen-Specific Regulation of Renin–Angiotensin System Genes in Epithelial Cells: A Comparative Study of SARS-CoV-2 Spike Protein N-Terminal Domain Fragment and Bacterial Lipopolysaccharide

**DOI:** 10.3390/pathogens15060593

**Published:** 2026-06-01

**Authors:** Aysegul Yılmaz, Seyhan Turk, Umit Yavuz Malkan, İbrahim Celalettin Haznedaroglu, Safiye Gocer, Sukru Volkan Ozguven, Can Turk

**Affiliations:** 1 Medical Microbiology Department, Faculty of Medicine, Lokman Hekim University, Ankara 06510, Türkiye; safiyegocer@lokmanhekim.edu.tr (S.G.); volkan.ozguven@lokmanhekim.edu.tr (S.V.O.); can.turk@lokmanhekim.edu.tr (C.T.); 2 Department of Biochemistry, Faculty of Pharmacy, Hacettepe University, Ankara 06230, Türkiye; seyhan.turk@hacettepe.edu.tr; 3 Department of Hematology, Faculty of Medicine, Hacettepe University, Ankara 06230, Türkiye; umit.malkan@hacettepe.edu.tr (U.Y.M.); i.celalettin.haznedaroglu@hacettepe.edu.tr (İ.C.H.)

**Keywords:** renin–angiotensin system, spike protein, lipopolysaccharide, epithelial barrier dysfunction, gene expression regulation

## Abstract

The renin–angiotensin system (RAS) regulates inflammation, tissue homeostasis, and barrier integrity in lung and colon epithelial cells. Beyond classical pathways, non-canonical components including angiotensin-converting enzyme 2 (ACE2), epidermal growth factor receptor (EGFR), insulin-like growth factor 2 receptor (IGF2R) and aminopeptidase N (ANPEP) are implicated in severe acute respiratory syndrome coronavirus 2 (SARS-CoV-2) infections and bacterial sepsis due to their roles in tissue repair and signaling. Despite their similar inflammatory and coagulopathic features, their impact on RAS-associated non-immune gene expression in epithelial tissues remains unclear. This study investigates the regulation of these targets in lung (BEAS-2B) and colon (CRL-1831) cells following exposure to recombinant SARS-CoV-2 spike protein N-terminal domain fragment (S1-NTD) and Pseudomonas aeruginosa-derived lipopolysaccharide (LPS). Cells were treated with 100 ng/mL of S1-NTD or LPS for 12–72 h. Viability was assessed via XTT assays, and molecular changes were analyzed through qRT-PCR and Western blotting. Both stimuli induced a time and dose-dependent decrease in metabolic activity. ACE2 was significantly downregulated in lung cells, while transient upregulation occurred in colon cells at 24 h. EGFR expression increased in colon cells following LPS exposure but decreased in lung cells after S1-NTD treatment. Both IGF2R and ANPEP were upregulated by S1-NTD in lung cells at 72 h, whereas colon cells showed earlier upregulation at 24–48 h. Our findings reveal that viral and bacterial stimuli elicit distinct, tissue-specific regulatory patterns in RAS-associated pathways. These alterations may contribute to epithelial barrier dysfunction and inflammation, highlighting these proteins as potential targets for managing secondary bacterial infections and inflammatory lung–gut complications in COVID-19.

## 1. Introduction

Coronavirus Disease 2019 (COVID-19), caused by the highly contagious severe acute respiratory syndrome coronavirus 2 (SARS-CoV-2), has profound implications for public health and global society [1]. In severe cases, critically ill patients frequently develop cytokine storms that resemble conditions observed in cases of sepsis and acute respiratory distress syndrome. COVID-19 patients also exhibit increased susceptibility to superinfections and secondary bacterial infections [2,3].

Lipopolysaccharide (LPS), a major structural component of the outer membranes of Gram-negative bacteria such as *P. aeruginosa*, triggers inflammatory responses through the activation of pathways such as TLR4, leading to cytokine release. Mouse models exposed to spike proteins and LPS demonstrate increased cytokine and chemokine levels, which are consistent with observations in the acute phases of COVID-19 infection [4].

The renin–angiotensin system (RAS) is a multifaceted regulator of epithelial cell function, modulating inflammation, tissue repair, and barrier integrity. While the classical RAS axis (ACE/Ang II/AT1R) promotes inflammation and vasoconstriction, angiotensin-converting enzyme 2 (*ACE2*) acts as a primary counter-regulator by cleaving angiotensin II into angiotensin (1–7), which exerts anti-inflammatory and cytoprotective effects. Beyond its enzymatic role, *ACE2* serves as the functional receptor for SARS-CoV-2. Another critical membrane-bound peptidase, alanyl aminopeptidase (*ANPEP/CD13*), works in tandem with *ACE2* to regulate bioactive peptides. In the intestinal and pulmonary epithelium, *ANPEP* and *ACE2* co-localize to maintain metabolic homeostasis and modulate local inflammatory responses; their dysregulation by pathogens can lead to the breakdown of epithelial barrier integrity [5,6]. Emerging evidence suggests that the impact of RAS dysregulation extends to non-canonical components, specifically epidermal growth factor receptor (*EGFR*) and insulin-like growth factor 2 receptor (*IGF2R*), which bridge RAS activity with tissue remodeling. *EGFR* is a key downstream mediator of the RAS axis; activation of the AT1 receptor by angiotensin II triggers ADAM17-mediated transactivation of *EGFR* [7]. This crosstalk is essential for epithelial restitution and repair, yet during SARS-CoV-2 infection, the spike–*ACE2* interaction can disrupt this signaling, leading to impaired lung and gut repair. Similarly, *IGF2R* serves as a multifunctional clearance receptor that modulates growth factor availability. Dysregulation of the RAS-induced inflammatory milieu can alter *IGF2R* expression, potentially hindering the clearance of apoptotic cells and contributing to fibrotic remodeling, as observed in severe COVID-19 and sepsis [8,9,10]. The SARS-CoV-2 virion consists of structural proteins, including envelope (E), matrix (M), nucleocapsid (N), and spike (S) proteins. The S protein comprises two subunits, S1 and S2, which are responsible for cell binding and membrane fusion, respectively [11]. Based on the convergence of inflammatory pathways in viral and bacterial insults, we hypothesized that the SARS-CoV-2 spike protein and *P. aeruginosa*-derived LPS disrupt epithelial barrier integrity through distinct but overlapping regulatory patterns of the expanded RAS network. Specifically, we posited that these pathogens differentially modulate the expression of non-canonical RAS components (*ACE2*, *EGFR*, *IGF2R* and *ANPEP*), thereby impairing the tissue capacity for anti-inflammatory signaling and repair in a tissue-specific manner. To test this hypothesis, we investigated how *Pseudomonas aeruginosa*-derived lipopolysaccharide (LPS) and SARS-CoV-2 spike protein N-terminal domain fragment (S1-NTD) modulate these molecules in lung (BEAS-2B) and colon (CRL-1831) epithelial cells, assessing their potential contribution to infection progression.

## 2. Materials and Methods

### 2.1. Reagents

All cell culture reagents utilized in this study were obtained from Capricorn Scientific, Ebsdorfergrund, Germany. Lyophilized LPS derived from *P. aeruginosa* (catalog number L9143) was sourced from Sigma-Aldrich, St. Louis, MO, USA and stored at 2–8 °C until required. Recombinant SARS-CoV-2 spike protein (NTD, His Tag) was purchased from Elabscience (Houston, TX, USA, Cat. No: PKSR030484). This specific recombinant fragment is derived from the ancestral Wuhan-Hu-1 (wild-type) lineage, corresponding to GenBank Accession #QHD43416.1, and precisely spans the amino acid sequence range from Gln14 to Asp290 expressed in HEK293 cells. For the sake of brevity, this N-terminal domain fragment of the spike protein is referred to as S1-NTD throughout the manuscript. A Cell Proliferation Kit II (XTT) was procured from Sigma-Aldrich, USA. Quantitative reverse transcription PCR (qRT-PCR) was performed using kits according to the manufacturers’ protocols. A.B.T.^TM^ qPCR Master Mix was acquired from ATLAS Biotechnology, Ankara, Turkey. All materials and equipment were either provided in sterile conditions or sterilized through autoclaving prior to use.

### 2.2. Cell Culture

Human colon epithelial cells (catalog number CRL-1831^TM^) and human lung epithelial cells (BEAS-2B; cat no: CRL-3588^TM^) were sourced from the American Type Culture Collection (ATCC, Manassas, VA, USA). The cells were cultivated in a modified Dulbecco’s Eagle medium enriched with L-Glutamine, 10% fetal bovine serum, and antibiotics (100 μg/mL penicillin and 100 μg/mL streptomycin). They were incubated under standard conditions of 5% CO_2_ at 37 °C. Experimental procedures commenced when the cells achieved a confluency of 70–80% [12,13].

### 2.3. Cell Proliferation

The XTT assay was employed to evaluate cell proliferation. Cells were seeded into 96-well plates at a density of 10^4^ cells per well and incubated for 24 h. After the initial incubation period, the cultures were exposed to a medium containing various concentrations (0.1, 1, 5, 10, 50 and 100 ng/mL) of S1-NTD and LPS for 12, 24, 48, and 72 h. Following these treatments, 50 µL of XTT reagent (1 mg/mL) was added to each well, and the plates were incubated for an additional 4 h. Absorbance was recorded at a wavelength of 450 nm using an ELISA microplate reader (BioTek Synergy H1, BioTek Instruments, Winooski, VT, USA) [14]. Cell viability was calculated as a percentage relative to the control group (untreated cells), which was normalized to 100%. All experimental groups were performed in three independent biological replicates to ensure statistical consistency.

### 2.4. Gene Expression Analysis

The effects of S1-NTD protein and LPS on the expression of RAS-related genes in CRL-1831 cells were assessed. The cells were treated with 100 ng/mL of LPS or 100 ng/mL of S1-NTD protein for 12, 24, 48 and 72 h [15,16]. Total RNA was extracted using TRIzol reagent (ABP Biosciences, Ankara, Turkey) and subjected to qPCR analysis utilizing the Lightcycler^®^ 96 system (Roche Diagnostic Systems, Indianapolis, IN, USA). Gene-specific primers were designed to target Glyceraldehyde 3-phosphate dehydrogenase (*GAPDH*), *ACE2*, *ANPEP*, *EGFR* and *IGF2R*.

The qPCR protocol consisted of an initial preheating step at 55 °C for 5 min and 95 °C for 5 min, followed by 40 cycles of denaturation at 95 °C for 15 s, annealing at 60 °C for 30 s, and extension at 72 °C for 1 min. Melting curve analysis was performed to ensure amplification specificity. Relative fold changes in gene expression were calculated using the comparative ΔΔCt method with target gene expression levels normalized to the internal reference gene, *GAPDH*. Each experiment, including the gene expression analyses, was performed in triplicate to ensure statistical reliability and reproducibility of the results. The primer sequences used in the study are provided in Table 1.

### 2.5. Western Blot

For Western blot analysis, CRL-1831 and BEAS-2B cells were cultured in 6-well plates at a density of 1 × 10^6^ cells per well and treated with 100 ng/mL of LPS or S1-NTD protein for 12, 24, 48, and 72 h at 37 °C. Post-incubation, the cells were lysed using ice-cold RIPA buffer to extract proteins. The protein concentrations were determined via the Bradford protein assay. Subsequently, equivalent protein amounts (10 μg) were resolved by SDS-PAGE on 10% gels and transferred to PVDF membranes.

Proteins were transferred to membranes using the iBlot 2 Gel Transfer Device (Invitrogen^TM^ IB21001, Thermo Fisher Scientific, Carlsbad, CA, USA). The membranes were then blocked for 1 h at room temperature with a blocking buffer. Following blocking, the membranes were incubated overnight at 4 °C with primary antibodies against ACE2 (1:3000; Cat# AF5165, Affinity Biotech, Cincinnati, OH, USA), EGFR (1:1000; Cat# M4112, Spring Bioscience, Pleasanton, CA, USA), IGF2R (1:750; Cat# DF8381, Affinity Biotech), and ANPEP (1:500; Cat# DF7445, Affinity Biotech). β-actin (1:3000; AF7018, Affinity Biotech) was used as the housekeeping protein for normalization. All primary antibodies were diluted in an antibody blocking solution according to the manufacturer’s instructions. Following a series of washes, the membranes were exposed to secondary antibodies (Goat Anti-Rabbit IgG (H + L) HRP; Cat#S0001, Affinity Biotech) at a 1:3000 dilution for 2 h at room temperature. Protein visualization was carried out using an ECL substrate, and densitometric analysis was performed using ImageJ version 1.53 analysis software, with β-actin serving as the loading control. All experiments were conducted in triplicate [17]. To maintain visual clarity and remove unrelated sample lanes processed concurrently on the same gel, digital images were cropped horizontally around the targeted molecular weights and vertically to isolate the relevant experimental groups. Original, uncropped images of all Western blots are provided in Supplementary Data S1.

### 2.6. Statistical Analysis

Each experiment was performed at least three times, and statistical analyses were conducted using GraphPad Prism software 8.01. Differences between two groups were analyzed using Student’s *t*-test, whereas differences among multiple groups were analyzed using ANOVA followed by Tukey’s post hoc test. Data are presented as mean ± SD, and differences were considered statistically significant at *p* < 0.05.

## 3. Results

### 3.1. Cell Viability in CRL-1831 Cells Treated with LPS and S1-NTD Protein

**Figure 1:**
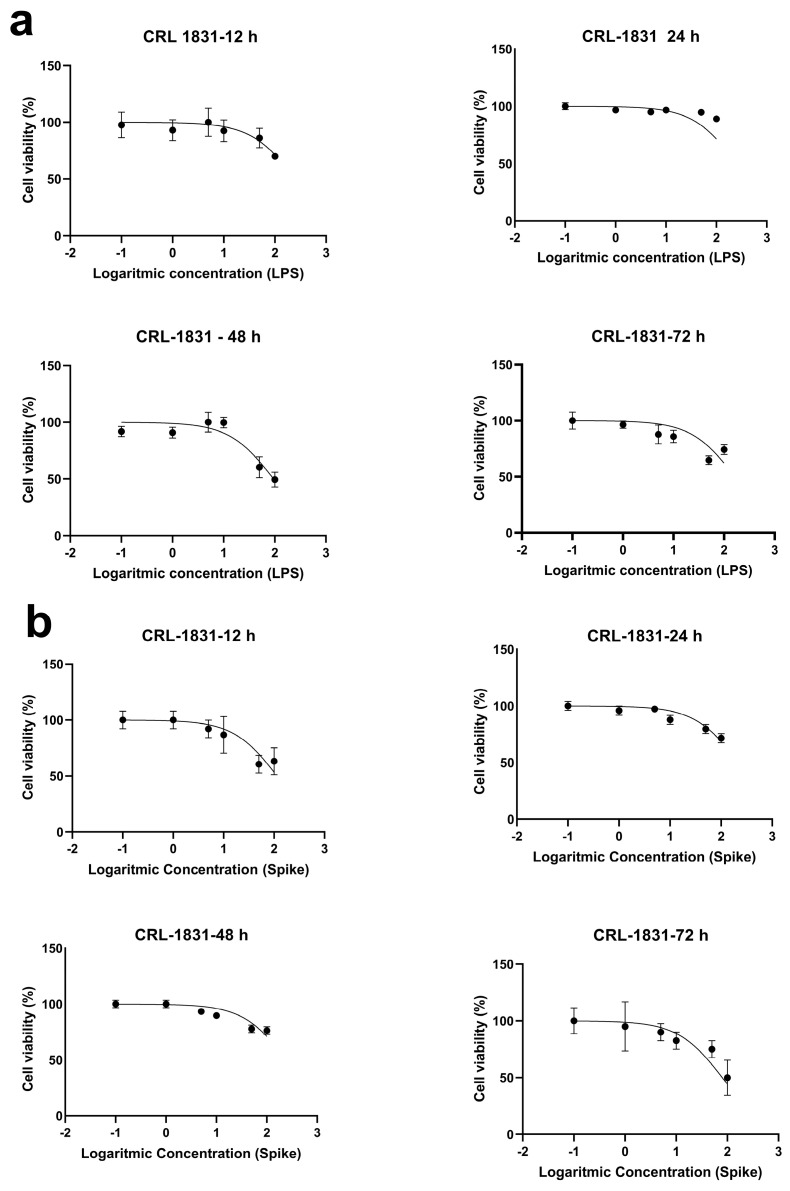
Dose-dependent viability assays in the CRL-1831 cell line. The cells were treated with various concentrations of LPS and S1-NTD (0.1, 1, 5, 10, 50, and 100 ng/mL). Dose–response curves of cytotoxicity of LPS (**a**) and S1-NTD protein (**b**) in CRL-1831 cells after 12 h, 24 h, 48 h, and 72 h incubation times. The *x*-axis represents increasing concentrations, with higher doses indicating progression through the treatment phases. It is observed that cell viability remained above 50% for all tested concentrations, indicating that both LPS and S1-NTD induced a dose-dependent decrease in metabolic activity under the experimental conditions. LPS, lipopolysaccharide; Spike, S1-NTD subunit fragment.

The impact of LPS and S1-NTD on epithelial cell viability was assessed across a range of concentrations and time intervals (Figure 1). Both treatments elicited a concentration and time-dependent reduction in metabolic activity, although cell viability remained above 50% across all tested conditions, indicating the preservation of homeostatic integrity under these specific experimental parameters.

In the early phases (12–24 h), LPS treatment did not significantly alter cell viability at lower doses. However, concentrations of 50 ng/mL and 100 ng/mL initiated a noticeable decline in metabolic rate compared to the control groups (Figure 1a). This inhibitory effect became statistically significant during the later stages (48–72 h), where a sustained reduction in viability was observed across all concentrations. Cells treated with the S1-NTD fragment exhibited a more immediate response compared to LPS, with a measurable reduction in viability occurring as early as 12 h and persisting through 24 and 48 h (Figure 1b). The most pronounced suppression was recorded at the 72 h time point, particularly following exposure to 100 ng/mL of S1-NTD. While both stimuli followed a comparable trajectory of dose-dependent suppression, S1-NTD appeared to induce an earlier metabolic shift than LPS. Notably, the maintenance of viability levels above the standard cytotoxic threshold throughout the 72 h period suggests that the observed effects are primarily characterized by metabolic suppression or a cytostatic response. These dose-dependent decreases in metabolic activity provided a suitable experimental framework to investigate the subsequent modulation of RAS-associated gene and protein expression without the confounding influence of widespread cell death.

### 3.2. Cell Viability in BEAS-2B Cells Treated with LPS and S1-NTD Protein

**Figure 2:**
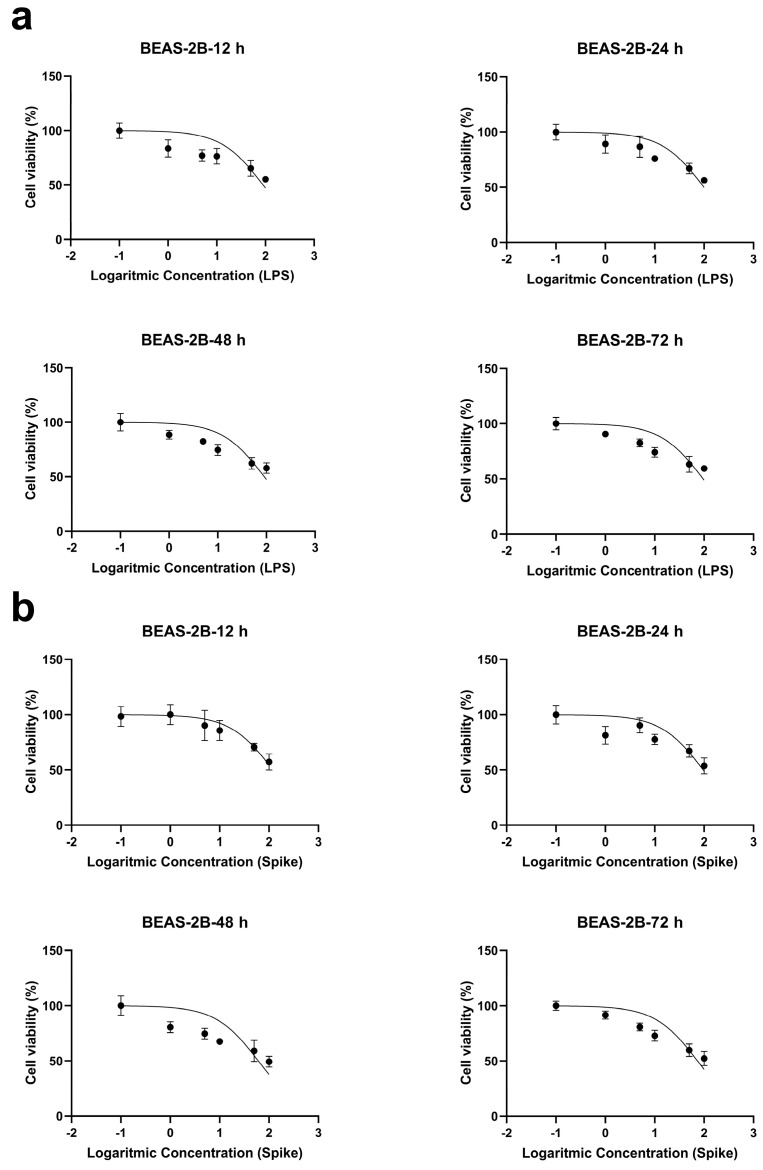
Dose-dependent viability assays in BEAS-2B cells. The cells were treated with various concentrations of LPS and S1-NTD (0.1, 1, 5, 10, 50, and 100 ng/mL). Dose–response curves of cytotoxicity of LPS (**a**) and S1-NTD protein (**b**) in BEAS-2B cells after 12 h, 24 h, 48 h, and 72 h incubation times. The *x*-axis represents increasing concentrations, with higher concentrations indicating progression through the treatment phases. It is observed that cell viability remained above 50% for all tested concentrations, indicating that both LPS and S1-NTD induced a dose-dependent decrease in metabolic activity under the experimental conditions. LPS, lipopolysaccharide; Spike, S1-NTD subunit fragment.

The metabolic response of lung epithelial (BEAS-2B) cells to LPS and S1-NTD treatments exhibited distinct temporal patterns compared to the colon (CRL-1831) line (Figure 2). While both stimuli induced dose-dependent fluctuations, the lung epithelium demonstrated a higher resilience to bacterial LPS while showing marked sensitivity to the viral S1-NTD fragment.

In contrast to the reductions observed in colon cells, LPS treatment in BEAS-2B cells did not result in statistically significant changes in cell viability across any of the tested time intervals or concentrations (Figure 2a). This suggests that at the applied concentrations, LPS does not compromise the metabolic integrity of the bronchial epithelium within the 72 h point. Conversely, S1-NTD treatment elicited a significant and progressive decline in cell viability (Figure 2b). Reductions began to stabilize between 24 and 48 h, even at lower concentrations such as 10 ng/mL, culminating in the most substantial metabolic suppression at 72 h with the 100 ng/mL concentration.

A comparative analysis of the two cell lines reveals that while S1-NTD acts as a potent metabolic suppressor in both lung and colon tissues, BEAS-2B cells exhibit a more specialized sensitivity to the viral protein fragment than to LPS. Consistent with the results obtained in CRL-1831 cells, viability remained well above the 50% threshold in all experimental groups. This confirms that the observed regulatory changes in the RAS network (*ACE2*, *EGFR*, *ANPEP*, and *IGF2R*) occur under conditions of dose-dependent metabolic suppression, further justifying the 100 ng/mL concentration for subsequent molecular analyses.

### 3.3. The Impact of S1-NTD Protein and LPS Treatment on RAS-Related Gene and Protein Expressions in CRL-1831 Cells

**Figure 3:**
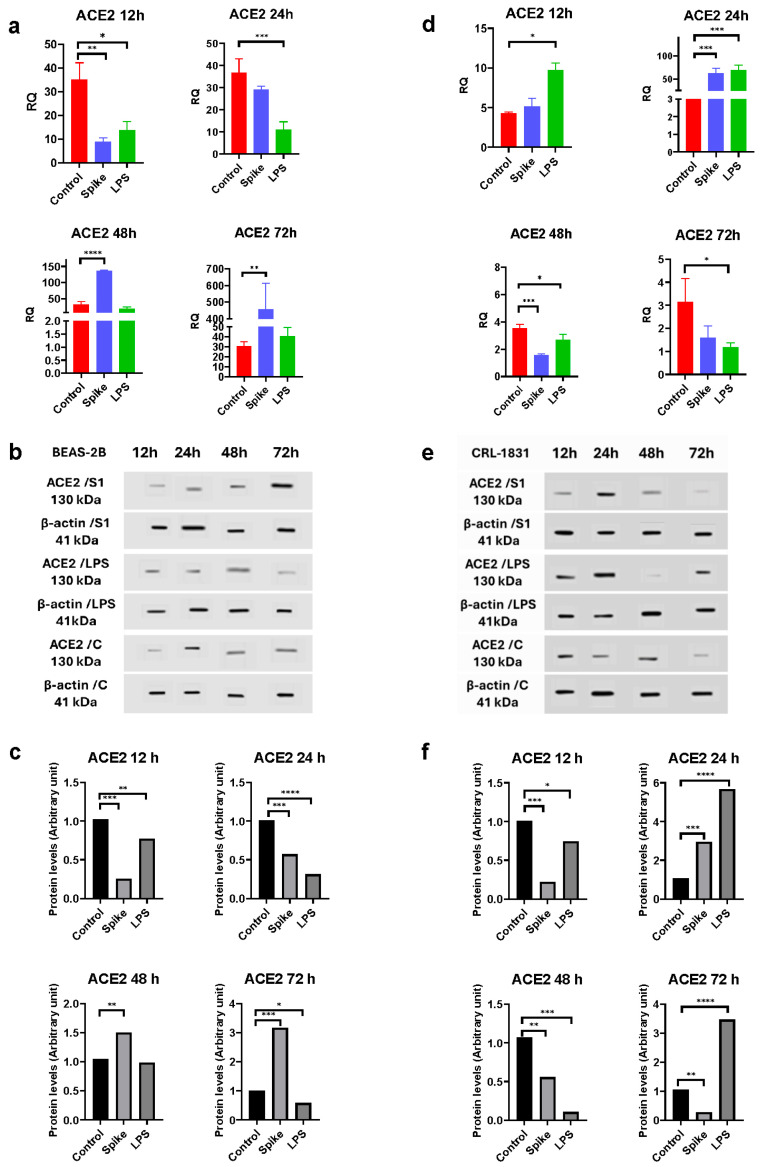
Time-dependent ACE2 mRNA and protein expression profiles in BEAS-2B and CRL-1831 cells. (**a**–**c**) Expression analysis in lung (BEAS-2B) and (**d**–**f**) intestinal (CRL-1831) epithelial cells following LPS (100 ng/mL) and S1-NTD protein (100 ng/mL) treatment over 72 h. (**a**,**d**) mRNA levels and (**b**,**c**,**e**,**f**) protein levels. S1-NTD protein and LPS induce significant, time-specific modulations of ACE2 across both cell lines. Statistical significance: * *p* < 0.05, ** *p* < 0.01, *** *p* < 0.001, and **** *p* < 0.0001. For protein normalization, β-actin served as the internal loading control. Note that the β-actin blots shown in (**b**,**e**) are the same as those in Figure 3, Figure 4, Figure 5 and Figure 6, as these targets were analyzed on the same membranes from identical experimental runs to ensure consistent normalization across multiple markers. The experimental groups were categorized as follows: the untreated control group (C) received only the basal culture medium for all time points, the S1-NTD group was treated with 100 ng/mL of the recombinant spike-NTD fragment (S1-NTD), and the LPS group was treated with 100 ng/mL of *P. aeruginosa*-derived lipopolysaccharide. LPS, lipopolysaccharide; Spike, S1-NTD subunit fragment.

**Figure 4:**
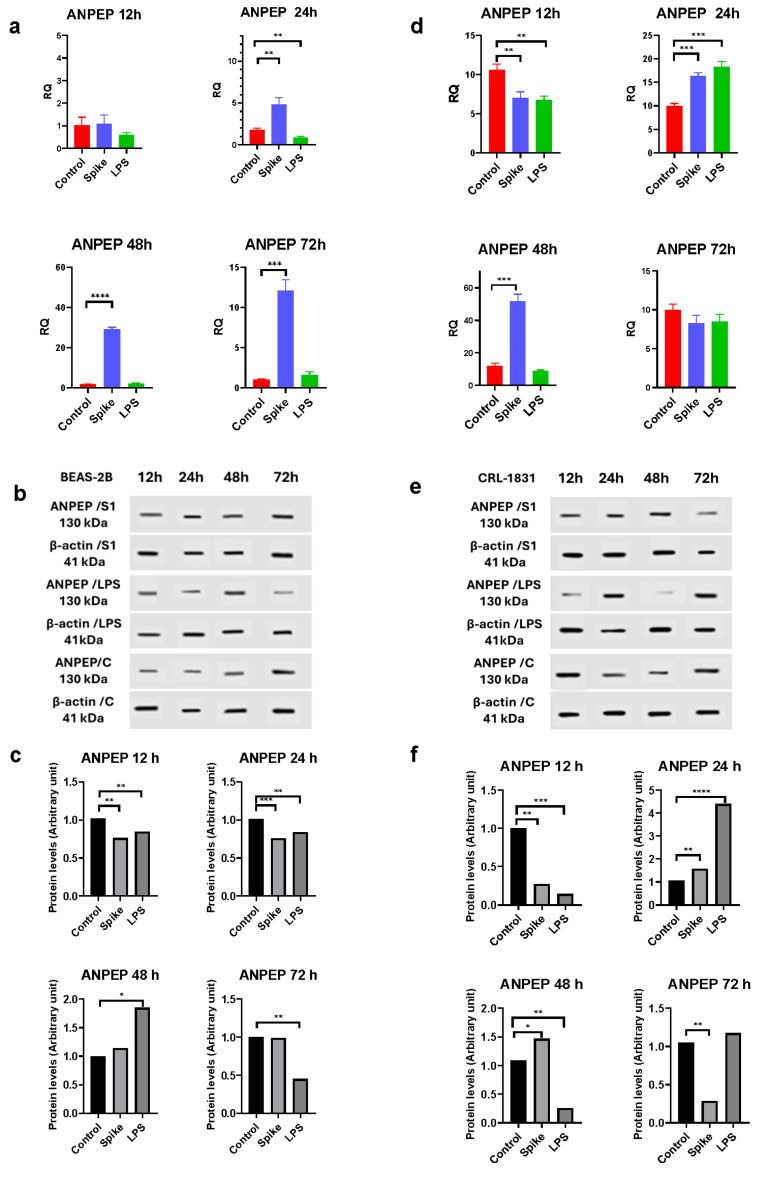
Comparative analysis of ANPEP mRNA and protein expression in lung and intestinal cells. (**a**–**c**) Time-course expression in BEAS-2B and (**d**–**f**) CRL-1831 cells following LPS and S1-NTD protein treatments (12–72 h). Panels (**a**,**d**) represent mRNA levels, while (**b**,**c**,**e**,**f**) show protein levels. S1-NTD protein significantly modulates ANPEP levels in a time-dependent manner across both cell lines. Statistical significance: * *p* < 0.05, ** *p* < 0.01, *** *p* < 0.001, and **** *p* < 0.0001. For protein normalization, β-actin served as the internal loading control. Note that the β-actin blots shown in (**b**,**e**) are the same as those in Figure 3, Figure 4, Figure 5 and Figure 6, as these targets were analyzed on the same membranes from identical experimental runs to ensure consistent normalization across multiple markers. The experimental groups were categorized as follows: the untreated control group (C) received only the basal culture medium for all time points, the S1-NTD group was treated with 100 ng/mL of the recombinant spike-NTD fragment (S1-NTD), and the LPS group was treated with 100 ng/mL of *P*. *aeruginosa*-derived lipopolysaccharide. LPS, lipopolysaccharide; Spike, S1-NTD subunit fragment.

In CRL-1831 cells treated with S1-NTD protein and LPS, *ACE2* expression levels increased at 12 and 24 h compared to the control group but decreased at 48 and 72 h (Figure 3). *ANPEP* expression levels were lower at 12 h in comparison to the control group; however, a significant increase in expression was observed at 24 h in both the S1-NTD protein- and LPS-treated groups (Figure 4).

**Figure 5:**
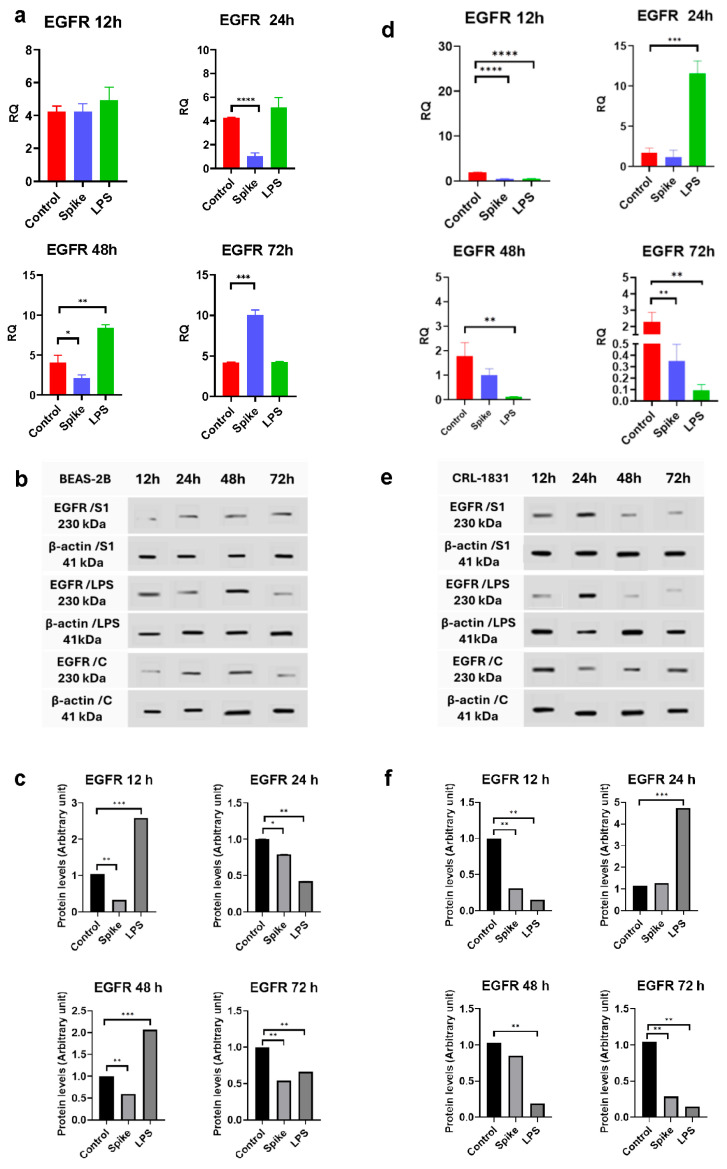
Modulation of EGFR mRNA and protein levels by LPS (100 ng/mL) and S1-NTD protein (100 ng/mL) in BEAS-2B and CRL-1831 cells. (**a**–**c**) Expression profiles in BEAS-2B cells and (**d**–**f**) CRL-1831 cells over a 72 h time course. Significant time-dependent fluctuations are observed, with S1-NTD protein notably inducing a decrease in EGFR protein levels across both cell lines. (**a**,**d**) mRNA expression; (**b**,**c**,**e**,**f**) protein expression. For protein normalization, β-actin served as the internal loading control. Note that the β-actin blots shown in (**b**,**e**) are the same as those in Figure 3, Figure 4, Figure 5 and Figure 6, as these targets were analyzed on the same membranes from identical experimental runs to ensure consistent normalization across multiple markers. Asterisks denote statistical significance (* *p* < 0.05, ** *p* < 0.01, *** *p* < 0.001, **** *p* < 0.0001) relative to the control. The experimental groups were categorized as follows: the untreated control group (C) received only the basal culture medium for all time points, the S1-NTD group was treated with 100 ng/mL of the recombinant spike-NTD fragment (S1-NTD), and the LPS group was treated with 100 ng/mL of *P. aeruginosa*-derived lipopolysaccharide. LPS, lipopolysaccharide; Spike, S1-NTD subunit fragment.

In CRL-1831 cells, a significant increase in *EGFR* expression was detected at 24 h, specifically following LPS treatment, while significantly lower expression levels were observed in the S1-NTD group compared to the control group. The same β-actin Western blot results were used to generate Figure 4 and Figure 5.

**Figure 6:**
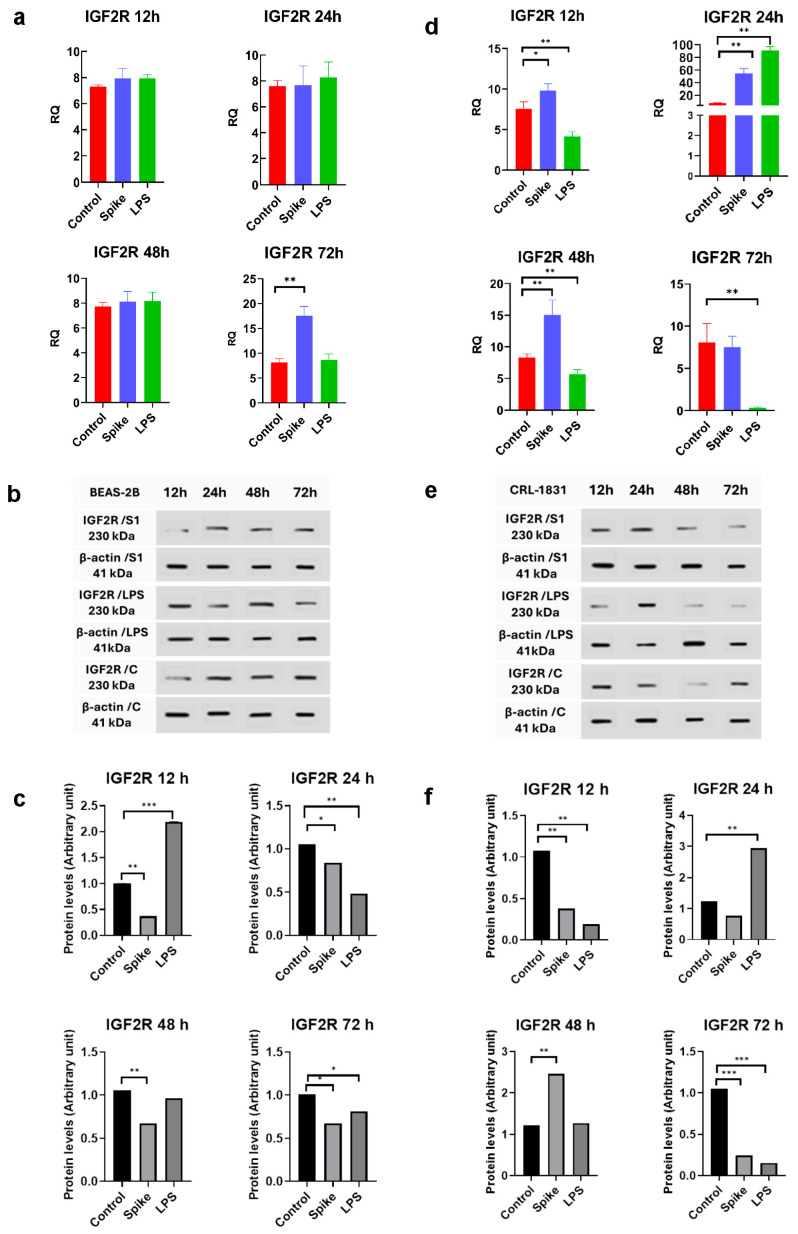
Impact of LPS (100 ng/mL) and S1-NTD protein (100 ng/mL) on IGF2R gene and protein expression profiles. (**a**–**c**) Differential expression in BEAS-2B cells and (**d**–**f**) CRL-1831 cells across 12, 24, 48, and 72 h time points. S1-NTD protein induces significant time-dependent upregulation of IGF2R mRNA while notably decreasing protein levels in BEAS-2B cells. (**a**,**d**) mRNA levels; (**b**,**c**,**e**,**f**) protein levels. Statistical significance: * *p* < 0.05, ** *p* < 0.01, and *** *p* < 0.001. For protein normalization, β-actin served as the internal loading control. Note that the β-actin blots shown in (**b**,**e**) are the same as those in Figure 3, Figure 4, Figure 5 and Figure 6, as these targets were analyzed on the same membranes from identical experimental runs to ensure consistent normalization across multiple markers. The experimental groups were categorized as follows: the untreated control group (C) received only the basal culture medium for all time points, the S1-NTD group was treated with 100 ng/mL of the recombinant spike-NTD fragment (S1), and the LPS group was treated with 100 ng/mL of *P. aeruginosa*-derived lipopolysaccharide. LPS, lipopolysaccharide; Spike, S1-NTD subunit fragment.

In the S1-NTD group, CRL-1831 cells exhibited significantly higher *IGF2R* expression levels only up to 48 h; by the 72 h time point, the expression levels had returned toward baseline, showing no significant difference compared to the control group. Meanwhile, in the LPS-treated groups, an increase was observed at 24 h, but lower *IGF2R* expression levels were detected during other time intervals (Figure 6).

### 3.4. The Impact of S1-NTD Protein and LPS Treatment on RAS-Related Gene and Protein Expressions in BEAS-2B Cells

In BEAS-2B cells, the groups treated with LPS exhibited significantly lower *ACE2* expression levels at 12 and 24 h. In contrast, the groups treated with S1-NTD protein demonstrated a significant increase in *ACE2* expression at 48 and 72 h (Figure 3). Similar to *ACE2* expression, *ANPEP* expression levels also significantly increased at 48 and 72 h in S1 protein-treated cells (Figure 4). For *EGFR* expression, significantly lower levels were detected in the S1-NTD protein-treated groups at 24 and 48 h time points, while a notable increase was observed at 72 h compared to the control group. In the LPS-treated groups, a significant increase in *EGFR* expression was observed only at 48 h (Figure 5). For *IGF2R* expression, an increase was detected at 72 h in the spike-treated groups, while no significant changes were observed in *IGF2R* expression in other time intervals for both the LPS- and S1-NTD protein-treated groups (Figure 6).

## 4. Discussion

*P. aeruginosa* is a Gram-negative opportunistic pathogen that possesses various virulence factors, such as LPS, that induce host immune responses [18]. In certain contexts, SARS-CoV-2 triggers robust inflammatory responses that may share common signaling pathways with bacterial-induced inflammation. Both bacterial sepsis and SARS-CoV-2 infections are associated with inflammatory and coagulopathic features, raising questions about their potential impacts on epithelial cells, particularly regarding the modulation of RAS-related genes [19].

Our previous study demonstrated significant alterations in the expression of *ACE2*, *ANPEP*, *EGFR* and *IGF2R* genes during the early (first phase) and spreading (second phase) stages of the disease [6]. It has been hypothesized that some aspects of the cellular response triggered by SARS-CoV-2 spike antigens may exhibit similarities to the responses induced by LPS. The inflammatory responses observed in alveolar and colon epithelial cells during severe clinical manifestations often parallel certain pathways activated by LPS [20]. Therefore, our study investigated the in vitro relationship between these genes and their differential expression patterns in response to S1-NTD and LPS. Our findings suggest that both the spike protein fragment and LPS can modulate the gene expression and protein levels of *ACE2*, *ANPEP*, *EGFR*, and *IGF2R* under the experimental conditions of this study.

While both viral and bacterial stimuli disrupted RAS-related gene expression, distinct patterns emerged. S1-NTD protein had a more sustained suppressive effect on *IGF2R* and *ACE2* in lung epithelial cells, whereas LPS initially upregulated *ACE2* in colon epithelial cells before decreasing expression over time. The upregulation of *ANPEP* by LPS but downregulation by S1-NTD protein may suggest differential modulation of epithelial permeability, which might influence susceptibility to co-infections.

Notably, *ACE2* has been recognized as the principal receptor facilitating SARS-CoV-2 infection in humans [21,22,23,24]. The interaction between SARS-CoV-2 spike and *ACE2* is a critical determinant of viral replication and disease severity. *ACE2* is also a critical regulator of the RAS, a hormonal cascade essential for cardiovascular and renal homeostasis. Within this system, angiotensinogen is cleaved by renin to form angiotensin I, which is subsequently converted to angiotensin II by *ACE* [25].

The modulations of *ACE2*, *ANPEP*, and *IGF2R* (AT2R-related signaling) observed in this study may potentially alter the balance of the expanded RAS axis. We observed a transient downregulation of *ACE2* in lung epithelial cells following S1-NTD and LPS exposure. Since *ACE2* is the primary enzyme responsible for the conversion of angiotensin II into the vasoprotective Ang-(1–7), its reduction could theoretically contribute to a localized accumulation of angiotensin II. Elevated Ang-II levels have been implicated in promoting inflammation, oxidative stress, and lung injury via the AT1R receptor [25].

The observed changes in *ANPEP* expression appear to be important for the downstream processing of peptides. *ANPEP* is responsible for the conversion of Ang-III into Ang-IV. Downregulation of *ANPEP*, as seen in certain time points of our study, could lead to a buildup of Ang-III, which, like Ang-II, may exert pressor and pro-inflammatory effects through AT1R, potentially exacerbating cellular damage. Angiotensin IV is a bioactive peptide that binds to the AT4R receptor. Our study tracked the expression of *IGF2R,* which shares complex signaling intersections with the RAS. A decrease in *ANPEP* activity could logically reduce the production of Ang-IV from Ang-III. Since Ang-IV is often associated with neuroprotective and anti-inflammatory roles in various tissues, its reduction, coupled with high Ang-II levels, might shift the cellular environment toward a more pro-pathogenic state [26].

Thuy et al. demonstrated that the spike protein binds to BEAS-2B cells, where *ACE2* is overexpressed under spike stimulation [27]. Our results demonstrate that both LPS and S1-NTD protein significantly downregulate *ACE2* expression, with a more pronounced reduction in lung epithelial cells at the 24 h time point. The differential regulation of these genes highlights distinct pathogen-specific mechanisms that may modulate epithelial susceptibility to infection and long-term tissue remodeling [28].

In the intestine, *ACE2* regulates the absorption of electrolytes and glucose. Abnormal *ACE2* expression may be associated with diarrhea, malnutrition, and other inflammatory diseases [29]. In a COVID-19 cohort, patients with diarrhea exhibited higher prevalence rates of detectable fecal viral RNA at admission [30]. Studies have reported *ACE2* expression in the human gastrointestinal system [31]. However, the expression patterns and intracellular localization of *ACE2* in the gastrointestinal system remain unclear. In our study, colon epithelial cells treated with spike protein showed increased *ACE2* expression and protein levels at 24 h, followed by a decline over subsequent time intervals.

An intriguing finding of our study is that the observed molecular alterations were induced by the isolated S1-NTD fragment rather than the full-length spike protein containing the Receptor-Binding Domain (RBD). Classically, SARS-CoV-2 cellular entry and associated signaling are driven by the RBD-*ACE2* interaction. However, growing evidence suggests that the NTD can independently trigger intracellular signaling cascades and facilitate viral attachment through alternative surface receptors. Evidence also indicates that regions of the spike protein outside the RBD, particularly the NTD, significantly contribute to viral antigenicity and host cell interactions [32,33]. Although the neutralizing activity of NTD-targeting neutralizing antibodies is generally reported to be weaker than that of their RBD-targeting counterparts, the NTD plays a critical role in immune evasion and alternative receptor engagement. Beyond its vaccine design implications, understanding how the isolated spike protein-NTD fragment independently modulates host epithelial responses is essential to fully elucidate the multifaceted pathogenesis of SARS-CoV-2 variants [34].

Notably, AXL has been identified as a candidate receptor that specifically interacts with the SARS-CoV-2 spike-NTD, promoting viral entry and inflammatory signaling in epithelial cells, independent of *ACE2* expression. AXL belongs to the family of TAM phosphatidylserine receptors and, similar to TIM family receptors, it has been reported to enhance cellular transduction efficiencies through an ‘apoptotic mimicry’ machinery. In our model, although S1-NTD exposure led to downstream dysregulation of RAS-associated genes like *ACE2* and *ANPEP*, this phenomenon may not be directly initiated by *ACE2* binding. Instead, the S1-NTD fragment might engage alternative host co-receptors such as AXL on epithelial cells. This engagement could transactivate downstream inflammatory networks or trigger receptor internalization, indirectly leading to the observed transient downregulation of cellular *ACE2* and *EGFR* levels. Future studies comprehensively profiling AXL expression and its direct inhibition are warranted to fully delineate this alternative, RBD-independent mechanistic pathway [35].

In a study comparing normal epithelial cells with LPS-treated colon epithelial cells, *ACE2* expression was found to increase in the LPS group at 24 h [9]. Similarly, our study observed elevated *ACE2* expression in the LPS group of colon epithelial cells at the 12 and 24 h time points. Wang et al. reported that *ACE2* expression remained close to control levels within the first 3–6 h, followed by downregulation over later time intervals [35]. While dysregulation of pulmonary ACE2 function is thought to play a role in respiratory diseases, including SARS-CoV, sepsis, and acid aspiration [36], its exact involvement in local inflammation and the underlying potential mechanisms remain incompletely explored and warrant further investigation [37].

*ANPEP*, or aminopeptidase N, plays a key role in inflammation and epithelial integrity. Our observations of an LPS-induced increase in *ANPEP* expression might be associated with processes such as bacterial adhesion or nutrient absorption. Conversely, the suppression observed following S1-NTD exposure could potentially indicate a disruption of normal epithelial functions. Given that *ANPEP*/CD13 is involved in leukocyte migration and inflammatory regulation, these findings could suggest that SARS-CoV-2 might modulate *ANPEP* activity to evade host immune responses, whereas LPS actively promotes its expression to sustain inflammation [38]. *ANPEP* has also been implicated in the gastrointestinal manifestations of COVID-19 [6].

The opposing regulation of *ANPEP* in lung and colon epithelial cells can be attributed to tissue-specific receptor expression and signaling contexts. In lung epithelial cells, high *ACE2* levels and associated interferon-responsive pathways may sensitize *ANPEP* to late-phase upregulation, potentially contributing to immune modulation and repair mechanisms [39]. Conversely, colon epithelial cells, which express lower *ACE2* but higher TLR4 and barrier-protective signaling, might downregulate *ANPEP* in response to S1-NTD exposure, potentially as a mechanism to preserve epithelial homeostasis and limit viral entry. It is possible that tissue-specific transcriptional programs, potentially involving NF-κB, interferon regulatory factors, and growth factor-related pathways, might drive these divergent responses [40]. Although not directly measured in this study, this integrated view tentatively suggests that the same viral stimulus could elicit opposite regulatory effects depending on the epithelial tissue type, potentially reconciling the seemingly contradictory observations reported here.

The *EGFR* gene plays a crucial role in cellular growth and proliferation. It has been observed to be upregulated in pulmonary cells infected with SARS-CoV-2, potentially contributing to the inflammation and damage seen in severe COVID-19 cases. Additionally, certain studies indicate that *EGFR*-targeting drugs may have therapeutic potential for COVID-19, although further investigation is required to substantiate this hypothesis. Recent studies have shown that LPS functionally transactivates *EGFR* in cholangiocarcinoma cells [41]. The differential expression of *EGFR* may further underscore these pathogen-specific responses.

Our study observed *EGFR* downregulation in lung cells following S1-NTD protein exposure at 24 and 48 h. Conversely, LPS-induced EGFR upregulation in colon cells aligns with previous reports suggesting that bacterial infections may promote epithelial proliferation and repair. These opposing responses might indicate that pathogen-specific modulation of *EGFR* signaling could potentially contribute to the divergent inflammatory profiles observed in the lung and gut [42].

The contrasting effects of S1-NTD protein and LPS on *EGFR* regulation can be explained by the fundamental differences in receptor usage and downstream signaling pathways between lung and colon epithelial cells. In lung cells, SARS-CoV-2 S1-NTD primarily engages *ACE2*, and the S1-NTD-*ACE2* interaction leads to internalization of the *ACE2-EGFR* complex, potentially resulting in reduced cell-surface *EGFR* availability and diminished repair signaling [43]. Spike activation also stimulates ADAM17, promoting *ACE2* shedding and indirectly suppressing *EGFR* transactivation. In contrast, LPS signals predominantly through TLR4, which is expressed at high levels in intestinal epithelium. LPS-TLR4 engagement induces Src-dependent *EGFR* transactivation, a well-characterized mucosal repair mechanism that promotes epithelial restitution and proliferation [44].

These differences are further amplified by tissue-specific receptor distributions: BEAS-2B cells may exhibit higher *ACE2* and lower TLR4 expression, whereas CRL-1831 cells display low *ACE2* but strong TLR4 activity. As a result, viral spike stimulation suppresses *EGFR* repair pathways in lung epithelial cells, while bacterial LPS activates *EGFR*-mediated repair in colon epithelium. This receptor-level divergence provides a plausible mechanistic hypothesis for the opposing effects noted in our findings.

The *IGF2R* gene plays a crucial role in regulating the activity of insulin-like growth factors, which are involved in processes such as cell growth and division. Downregulation of *IGF2R* has been observed in lung cells infected with SARS-CoV-2, a change that may contribute to the lung inflammation and damage characteristic of severe COVID-19 cases. While some studies propose that *IGF2R* may participate in the immune response to viral infections, further investigations are required to elucidate its exact role [45]. *IGF2R* suppression by S1-NTD protein in lung epithelial cells could align with prior studies linking *IGF2R* downregulation to impaired immune responses in severe COVID-19. It has been suggested that reduced *IGF2R* levels might hinder the clearance of damaged cells, potentially contributing to persistent inflammation and fibrotic remodeling.

In contrast, the transient upregulation of *IGF2R* in colon cells exposed to LPS suggests a context-dependent role in bacterial infections, potentially mediating protective or pathogenic effects depending on the inflammatory milieu. Interestingly, high expression of the *IGF2R* gene has been reported in pediatric patients, potentially contributing to the milder clinical manifestations of COVID-19 observed in this population [7].

Importantly, the opposing responses observed between lung and colon epithelial cells might be attributed to fundamental differences in their baseline inflammatory signatures, receptor expression profiles and tissue-specific signaling networks. Airway epithelial cells generally display stronger TLR2/TLR4-mediated NF-κB activation and heightened basal inflammatory tone, which amplifies viral antigen-induced cytokine pathways [46]. In contrast, colonic epithelial cells possess more robust interferon-related and barrier-protective signaling, shaped by continuous microbial exposure [47]. These intrinsic programs can modulate the effects of LPS and SARS-CoV-2 spike protein differently, leading to tissue-dependent responses. Moreover, variable *ACE2*, *ANPEP*, *EGFR* and *IGF2R* expression across the lung and intestine further contributes to divergent signaling outcomes. Prior studies have shown that SARS-CoV-2 spike preferentially suppresses *ACE2* and *IGF2R* signaling in lung epithelial cells [48], whereas LPS activates *EGFR* and *ANPEP* in intestinal epithelium as part of epithelial repair and inflammatory adaptation [5,41]. Therefore, the contrasting patterns observed in our results likely reflect intrinsic, tissue-specific immunological programming and receptor distribution, providing a unifying biological explanation for the divergent responses highlighted in this study.

The RAS plays a pivotal role in processes such as inflammation, cell proliferation, angiogenesis, and fibrosis [49]. Recent studies have highlighted that epithelial tissues possess an actively regulated local RAS, which responds dynamically to inflammatory, microbial, and viral stimuli. In airway epithelium, SARS-CoV-2 spike protein has been shown to modulate components of the *ACE2*/Ang-(1–7)/Mas axis, altering epithelial barrier function and cytokine signaling [50]. Similarly, bacterial components such as LPS induce shifts toward the pro-inflammatory ACE/Ang II/AT1R axis, promoting epithelial permeability, oxidative stress, and tissue remodeling [51]. These studies may collectively suggest that the epithelial RAS behaves as a context-dependent regulatory network rather than a static pathway. By comparing the divergent effects of S1-NTD protein and LPS on *ACE2*, *ANPEP*, *IGF2R*, and *EGFR* expression across two epithelial cell types, our findings align with and extend this literature, suggesting that distinct pathogens differentially reprogram epithelial RAS signaling. Whether the epithelial RAS has potential as a biomarker or therapeutic target remains incompletely understood; therefore, it is advised that future studies rigorously explore this possibility [10]. While both pathogens disrupt RAS-related gene expression, they appear to exhibit distinct regulatory mechanisms, which may contribute to tissue-specific damage and inflammation.

The sustained *ACE2* downregulation in lung epithelial cells following S1-NTD protein exposure may exacerbate pulmonary injury in COVID-19, whereas the transient upregulation in colon cells may contribute to gut barrier dysfunction and diarrhea. Conversely, LPS-induced *EGFR* activation in colon cells highlights a potential compensatory mechanism to preserve epithelial integrity. The differential regulation of *IGF2R* and *ANPEP* by viral and bacterial factors further suggests that SARS-CoV-2 and bacterial infections employ distinct strategies to alter epithelial homeostasis [52].

A primary limitation of this study is the use of the isolated S1-NTD fragment rather than a live SARS-CoV-2 infection model. While stimulation with recombinant spike fragments is a widely accepted approach to investigate receptor-mediated signaling, it does not fully recapitulate the biological complexity of a viral infection, which involves multiple viral proteins, replication cycles and intracellular pattern-recognition pathways. Therefore, the responses observed here likely represent only a specific component of the broader host reaction. Although a wide range of concentrations was initially screened, gene and protein expression analyses were conducted using a single-dose model (100 ng/mL). Consequently, potential dose-dependent variations in molecular expression patterns remain to be fully elucidated in future studies. Due to the inherent sample size limitations of independent biological replicates (*n* = 3) typical in in vitro assays, formal normality tests (such as the Shapiro–Wilk test) have limited statistical power to accurately detect data distribution. However, because the experiments were performed under highly standardized and controlled cell culture conditions to minimize external variability, data distribution was carefully evaluated, and parametric analyses were deemed appropriate for detecting significant differences.

Given the molecular similarities between S1-NTD protein and LPS-induced inflammation, further research is needed to explore the precise pathways by which these agents amplify immune responses and disrupt epithelial barrier function. These findings provide preliminary insights into the differential regulation of epithelial function and suggest potential mechanisms underlying COVID-19 complications. Future studies incorporating in vivo models could provide deeper insights into the clinical implications of these findings and potential therapeutic interventions.

## Figures and Tables

**Table 1:** RNA primers used in gene expression analysis.

Gene Symbol	Forward	Reverse
*ACE2*	TGGGTCTTCAGTGCTCTCAG	ACCCCACATATCACCAAGCA
*ANPEP*	GGCCCCATGAAGAACTACCT	TCAGCCTCATTGACCAGTGT
*GAPDH*	CCAGAACATCATCCCTGCCT	CCTGCTTCACCACCTTCTTG
*EGFR*	TCATGCTCTACAACCCCACC	GCACTTCTTACACTTGCGGA
*IGF2R*	CTTTGACAGCGAGAATCCCG	TCACTGTTTCCCTCCTCTCC

## Data Availability

The data generated in the present study may be requested from the corresponding author.

## Supplementary Materials

The following supporting information can be downloaded at https://www.mdpi.com/article/10.3390/pathogens15060593/s1, Supplementary Data S1: Original western blot images.

## References

[1] Lamers M.M., Haagmans B.L. (2022). SARS-CoV-2 pathogenesis. Nat. Rev. Microbiol..

[2] Hu B., Guo H., Zhou P., Shi Z.L. (2021). Characteristics of SARS-CoV-2 and COVID-19. Nat. Rev. Microbiol..

[3] Mohamadian M., Chiti H., Shoghli A., Biglari S., Parsamanesh N., Esmaeilzadeh A. (2021). COVID-19: Virology, biology and novel laboratory diagnosis. J. Gene Med..

[4] Bertani B., Ruiz N. (2018). Function and Biogenesis of Lipopolysaccharides. EcoSal Plus.

[5] Lang C., Huang B., Chen Y., He Z. (2025). The role of the classical renin-angiotensin system and angiotensin-converting enzyme 2/Ang(1-7)/Mas axis in pulmonary fibrosis. Front. Med..

[6] López-Cortés G.I., Palacios-Pérez M., Hernández-Aguilar M.M., Veledíaz H.F., José M.V. (2023). Human Coronavirus Cell Receptors Provide Challenging Therapeutic Targets. Vaccines.

[7] Gekle M., Dubourg V., Schwerdt G., Benndorf R.A., Schreier B. (2023). The role of EGFR in vascular AT1R signaling: From cellular mechanisms to systemic relevance. Biochem. Pharmacol..

[8] Şenel E., Türk S., Malkan Ü.Y., Peker M.Ç., Türk C., Güner H.R., Uçar G., Izdeş S., Kayaaslan B., Bayhan G.İ. (2023). Pathobiological alterations affecting the distinct clinical courses of pediatric versus adult COVID-19 syndrome. Turk. J. Med. Sci..

[9] Turk C., Turk S., Malkan U.Y., Haznedaroglu I.C. (2020). Three critical clinicobiological phases of the human SARS-associated coronavirus infections. Eur. Rev. Med. Pharmacol. Sci..

[10] Krenn K., Kraft F., Mandroiu L., Tretter V., Reindl-Schwaighofer R., Clement T., Domenig O., Vossen M.G., Riemann G., Poglitsch M. (2025). Renin–angiotensin–aldosterone system activation in plasma as marker for prognosis in critically ill patients with COVID-19: A prospective exploratory study. Ann. Intensive Care.

[11] Kunvariya A.D., Dave S.A., Modi Z.J., Patel P.K., Sagar S.R. (2023). Exploration of multifaceted molecular mechanism of angiotensin-converting enzyme 2 (ACE2) in pathogenesis of various diseases. Heliyon.

[12] Yang K., Wang Y. (2024). Dandelion root extracts and taraxasterol inhibit LPS-induced colorectal cancer cell viability by blocking TLR4-NFκB-driven ACE2 and TMPRSS2 pathways. Exp. Ther. Med..

[13] Baysal I., Örsten S. (2021). Evaluation of the effect of hydatid cyst fluid on the apoptosis pathway in BEAS-2B and A549 cell lines. Mikrobiyol. Bul..

[14] Lee D.K., Jang S., Kim M.J., Kim J.H., Chung M.J., Kim K.J., Ha N.J. (2008). Anti-proliferative effects of Bifidobacterium adolescentis SPM0212 extract on human colon cancer cell lines. BMC Cancer.

[15] Kang H., Kim D., Min K., Park M., Kim S.-H., Sohn E.-J., Choi B.-H., Hwang I. (2022). Recombinant proteins of spike protein of SARS-CoV-2 with the Omicron receptor-binding domain induce production of highly Omicron-specific neutralizing antibodies. Clin. Exp. Vaccine Res..

[16] Wang W.-T., Zhang Y.-Y., Li Z.-R., Li J.-M., Deng H.-S., Li Y.-Y., Yang H.-Y., Lau C.C., Yao Y.-J., Pan H.-D. (2024). Syringic acid attenuates acute lung injury by modulating macrophage polarization in LPS-induced mice. Phytomedicine.

[17] Chen Z., Wu H., Fan W., Zhang J., Yao Y., Su W., Wang Y., Li P. (2022). Naringenin suppresses BEAS-2B-derived extracellular vesicular cargoes disorder caused by cigarette smoke extract thereby inhibiting M1 macrophage polarization. Front. Immunol..

[18] Huszczynski S.M., Lam J.S., Khursigara C.M. (2020). The role of Pseudomonas aeruginosa lipopolysaccharide in bacterial pathogenesis and physiology. Pathogens.

[19] Petruk G., Puthia M., Petrlova J., Samsudin F., Strömdahl A.-C., Cerps S., Uller L., Kjellström S., Bond P.J., Schmidtchen A. (2020). SARS-CoV-2 spike protein binds to bacterial lipopolysaccharide and boosts proinflammatory activity. J. Mol. Cell Biol..

[20] Samsudin F., Raghuvamsi P., Petruk G., Puthia M., Petrlova J., MacAry P., Anand G.S., Bond P.J., Schmidtchen A. (2023). SARS-CoV-2 spike protein as a bacterial lipopolysaccharide delivery system in an overzealous inflammatory cascade. J. Mol. Cell Biol..

[21] Lan J., Ge J., Yu J., Shan S., Zhou H., Fan S., Zhang Q., Shi X., Wang Q., Zhang L. (2020). Structure of the SARS-CoV-2 spike receptor-binding domain bound to the ACE2 receptor. Nature.

[22] Wang Q., Zhang Y., Wu L., Niu S., Song C., Zhang Z., Lu G., Qiao C., Hu Y., Yuen K.Y. (2020). Structural and Functional Basis of SARS-CoV-2 Entry by Using Human ACE2. Cell.

[23] Gheblawi M., Wang K., Viveiros A., Nguyen Q., Zhong J.-C., Turner A.J., Raizada M.K., Grant M.B., Oudit G.Y. (2020). Angiotensin-Converting Enzyme 2: SARS-CoV-2 Receptor and Regulator of the Renin-Angiotensin System: Celebrating the 20th Anniversary of the Discovery of ACE2. Circ. Res..

[24] Luo D., Bai M., Zhang W., Wang J. (2024). The possible mechanism and research progress of ACE2 involved in cardiovascular injury caused by COVID-19: A review. Front. Cardiovasc. Med..

[25] Valentini A., Heilmann R.M., Kühne A., Biagini L., De Bellis D., Rossi G. (2025). The Renin-Angiotensin-Aldosterone System (RAAS): Beyond Cardiovascular Regulation. Vet. Sci..

[26] Gagliardi S., Hotchkin T., Tibebe H., Hillmer G., Marquez D., Izumi C., Chang J., Diggs A., Ezaki J., Suzuki Y.J. (2025). The Renin-Angiotensin System Modulates SARS-CoV-2 Entry via ACE2 Receptor. Viruses.

[27] Thuy P.X., Bao T.D.D., Moon E.Y. (2022). Ursodeoxycholic acid ameliorates cell migration retarded by the SARS-CoV-2 spike protein in BEAS-2B human bronchial epithelial cells. Biomed. Pharmacother..

[28] Ma J., Zhu Z., Yishajiang Y., Alarjani K.M., Hong L., Luo L. (2023). Role of gut microbiota and inflammatory factors in acute respiratory distress syndrome: A Mendelian randomization analysis. Front. Microbiol..

[29] Hashimoto T., Perlot T., Rehman A., Trichereau J., Ishiguro H., Paolino M., Sigl V., Hanada T., Hanada R., Lipinski S. (2012). ACE2 links amino acid malnutrition to microbial ecology and intestinal inflammation. Nature.

[30] Xiao F., Tang M., Zheng X., Liu Y., Li X., Shan H. (2020). Evidence for Gastrointestinal Infection of SARS-CoV-2. Gastroenterology.

[31] Qi F., Qian S., Zhang S., Zhang Z. (2020). Single cell RNA sequencing of 13 human tissues identify cell types and receptors of human coronaviruses. Biochem. Biophys. Res. Commun..

[32] Chi X., Yan R., Zhang J., Zhang G., Zhang Y., Hao M., Zhang Z., Fan P., Dong Y., Yang Y. (2020). A neutralizing human antibody binds to the N-terminal domain of the Spike protein of SARS-CoV-2. Science.

[33] Wang Z., Muecksch F., Cho A., Gaebler C., Hoffmann H.-H., Ramos V., Zong S., Cipolla M., Johnson B., Schmidt F. (2022). Analysis of memory B cells identifies conserved neutralizing epitopes on the N-terminal domain of variant SARS-CoV-2 spike proteins. Immunity.

[34] Li W., Liu C., Li Y., Gui Q., Cheng L., Fan Q., Zhou B., Wang H., Ge X., Zhang Z. (2026). A SARS-CoV-2 variant-induced NTD-targeting antibody enhances viral infection via a distinctive binding mode. PLoS Pathog..

[35] Wang S., Qiu Z., Hou Y., Deng X., Xu W., Zheng T., Wu P., Xie S., Bian W., Zhang C. (2021). AXL is a candidate receptor for SARS-CoV-2 that promotes infection of pulmonary and bronchial epithelial cells. Cell Res..

[36] Sodhi C.P., Wohlford-Lenane C., Yamaguchi Y., Prindle T., Fulton W.B., Wang S., McCray P.B., Chappell M., Hackam D.J., Jia H. (2018). Attenuation of pulmonary ACE2 activity impairs inactivation of des-arg9 bradykinin/BKB1R axis and facilitates LPS-induced neutrophil infiltration. Am. J. Physiol.—Lung Cell Mol. Physiol..

[37] Ahmad S., Ahmed M.M., Hasan P.M.Z., Sharma A., Bilgrami A.L., Manda K., Ishrat R., Syed M.A. (2020). Identification and validation of potential miRNAs, as biomarkers for sepsis and associated lung injury: A network-based approach. Genes.

[38] Kim J.-H., Afridi R., Cho E., Yoon J.H., Lim Y.-H., Lee H.-W., Ryu H., Suk K. (2022). Soluble ANPEP Released from Human Astrocytes as a Positive Regulator of Microglial Activation and Neuroinflammation: Brain Renin-Angiotensin System in Astrocyte-Microglia Crosstalk. Mol. Cell Proteom..

[39] Ziegler C.G.K., Allon S.J., Nyquist S.K., Mbano I.M., Miao V.N., Tzouanas C.N., Cao Y., Yousif A.S., Bals J., Hauser B.M. (2020). SARS-CoV-2 Receptor ACE2 Is an Interferon-Stimulated Gene in Human Airway Epithelial Cells and Is Detected in Specific Cell Subsets across Tissues. Cell.

[40] Okumura R., Takeda K. (2017). Roles of intestinal epithelial cells in the maintenance of gut homeostasis. Exp. Mol. Med..

[41] Trussoni C.E., Tabibian J.H., Splinter P.L., O’Hara S.P. (2015). Lipopolysaccharide (LPS)-induced biliary epithelial cell NRas activation requires Epidermal Growth Factor Receptor (EGFR). PLoS ONE.

[42] Hardbower D.M., Singh K., Asim M., Verriere T.G., Olivares-Villagómez D., Barry D.P., Allaman M.M., Washington M.K., Peek R.M., Piazuelo M.B. (2016). EGFR regulates macrophage activation and function in bacterial infection. J. Clin. Invest..

[43] Hoffmann M., Kleine-Weber H., Schroeder S., Krüger N., Herrler T., Erichsen S., Schiergens T.S., Herrler G., Wu N.-H., Nitsche A. (2020). SARS-CoV-2 Cell Entry Depends on ACE2 and TMPRSS2 and Is Blocked by a Clinically Proven Protease Inhibitor. Cell.

[44] Heurich A., Hofmann-Winkler H., Gierer S., Liepold T., Jahn O., Pöhlmann S. (2014). TMPRSS2 and ADAM17 cleave ACE2 differentially and only proteolysis by TMPRSS2 augments entry driven by the severe acute respiratory syndrome coronavirus spike protein. J. Virol..

[45] Zolov S.N., Imai H., Losiewicz M.K., Singh R.S.J., Fort P.E., Gardner T.W. (2021). Insulin-like growth factor-2 regulates basal retinal insulin receptor activity. J. Biol. Chem..

[46] Sidletskaya K., Vitkina T., Denisenko Y. (2020). The Role of Toll-Like Receptors 2 and 4 in the Pathogenesis of Chronic Obstructive Pulmonary Disease. Int. J. Chron. Obs. Pulmon Dis..

[47] Dou X., Han J., Ma Q., Cheng B., Shan A., Gao N., Yang Y. (2018). TLR2/4-mediated NF-κB pathway combined with the histone modification regulates β-defensins and interleukins expression by sodium phenyl butyrate in porcine intestinal epithelial cells. Food Nutr. Res..

[48] Sui Y., Li J., Venzon D.J., Berzofsky J.A. (2021). SARS-CoV-2 Spike Protein Suppresses ACE2 and Type I Interferon Expression in Primary Cells From Macaque Lung Bronchoalveolar Lavage. Front. Immunol..

[49] Triebel H., Castrop H. (2024). The renin angiotensin aldosterone system. Pflug. Arch. Eur. J. Physiol..

[50] Magazine R., Chogtu B., Bhat A. (2023). Role of Angiotensin Converting Enzyme-2 and its modulation in disease: Exploring new frontiers. Med. Pharm. Rep..

[51] Haliga R.E., Cojocaru E., Sîrbu O., Hrițcu I., Alexa R.E., Haliga I.B., Șorodoc V., Coman A.E. (2025). Immunomodulatory Effects of RAAS Inhibitors: Beyond Hypertension and Heart Failure. Biomedicines.

[52] Al-Ani B., ShamsEldeen A.M., Kamar S.S., Haidara M.A., Al-Hashem F., Alshahrani M.Y., Al-Hakami A.M., Kader D.H.A., Maarouf A. (2022). Lipopolysaccharide induces acute lung injury and alveolar haemorrhage in association with the cytokine storm, coagulopathy and AT1R/JAK/STAT augmentation in a rat model that mimics moderate and severe COVID-19 pathology. Clin. Exp. Pharmacol. Physiol..

