# Supplementary figures and images for: Pathogen-Specific Regulation of Renin–Angiotensin System Genes in Epithelial Cells: A Comparative Study of SARS-CoV-2 Spike Protein N-Terminal Domain Fragment and Bacterial Lipopolysaccharide
Source: Pathogens. 2026 Jun 1;15(6):593. doi: 10.3390/pathogens15060593 (PMC13304810; doi:10.3390/pathogens15060593)

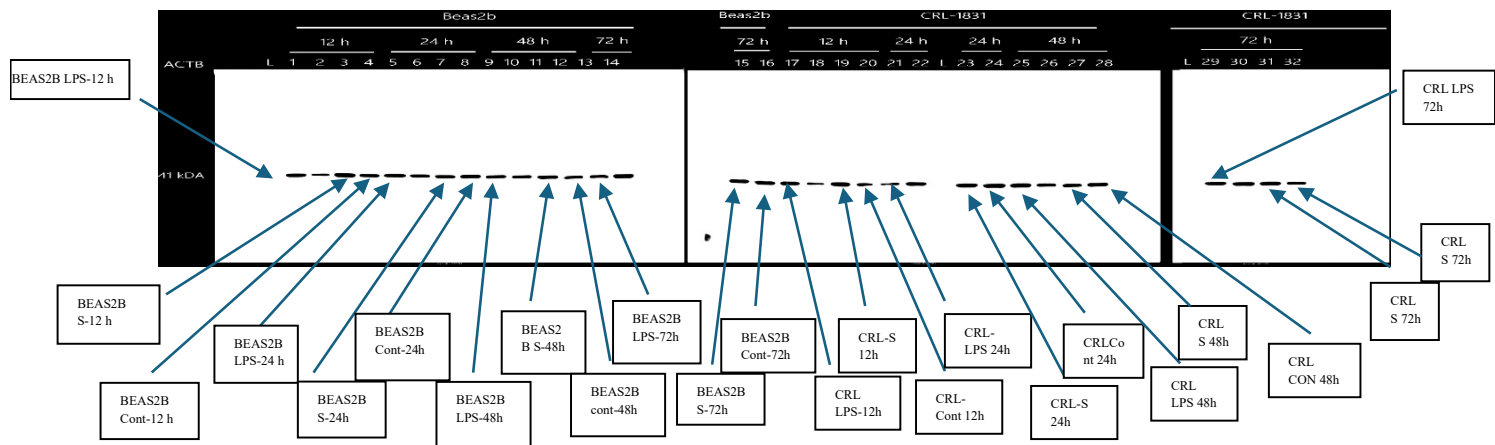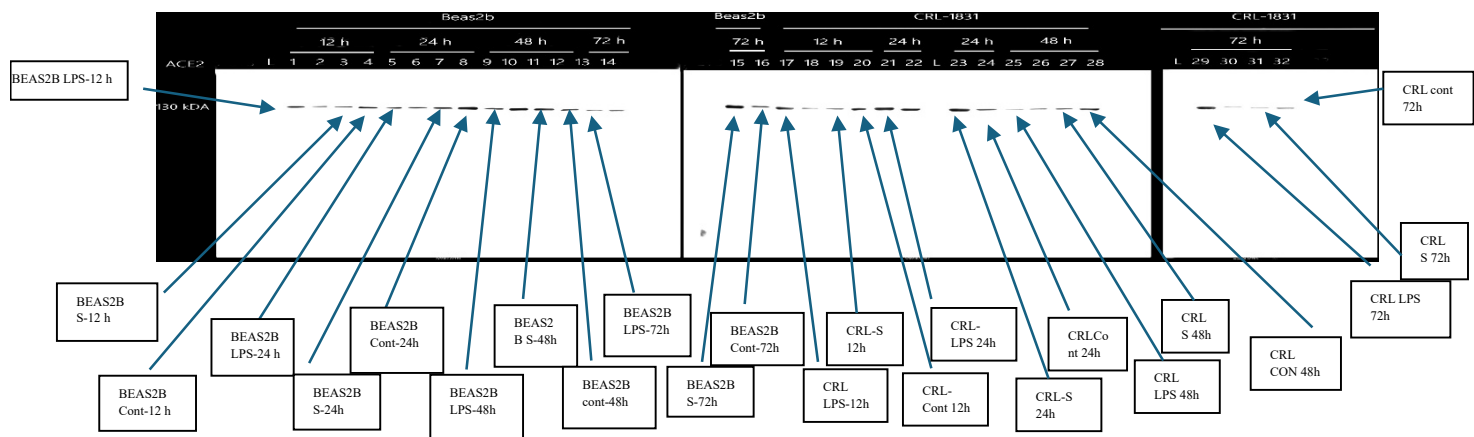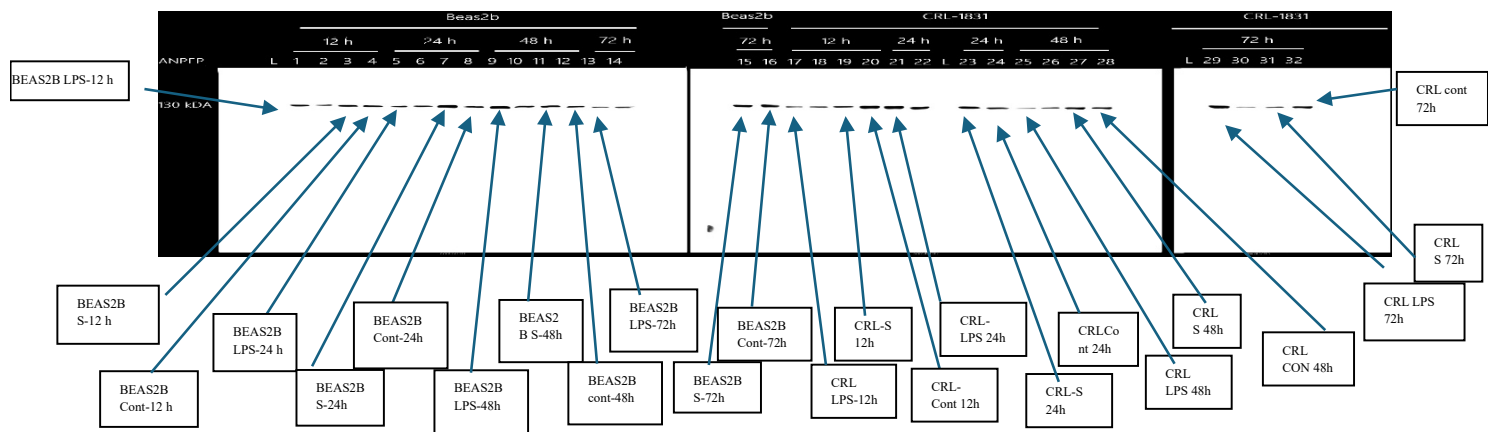

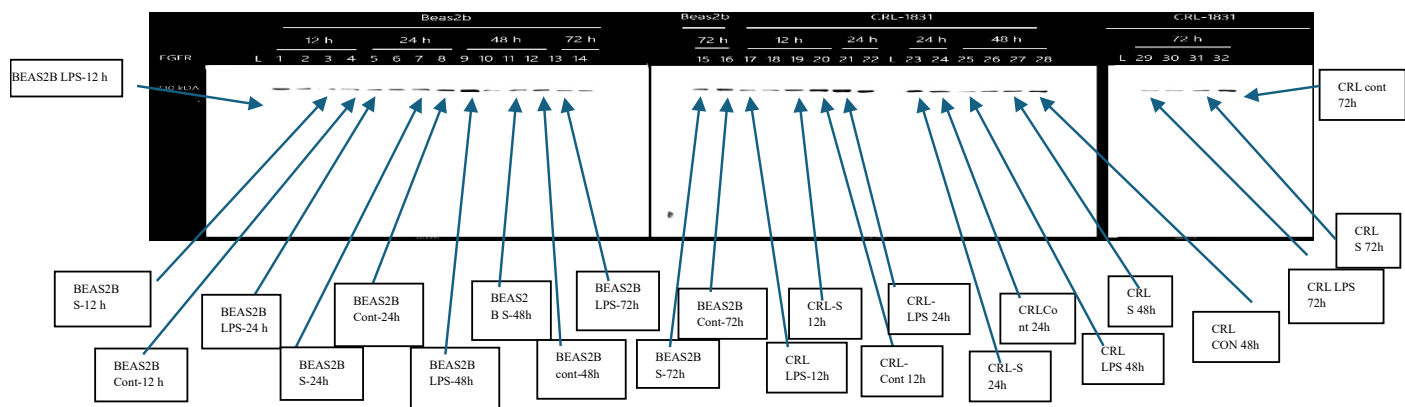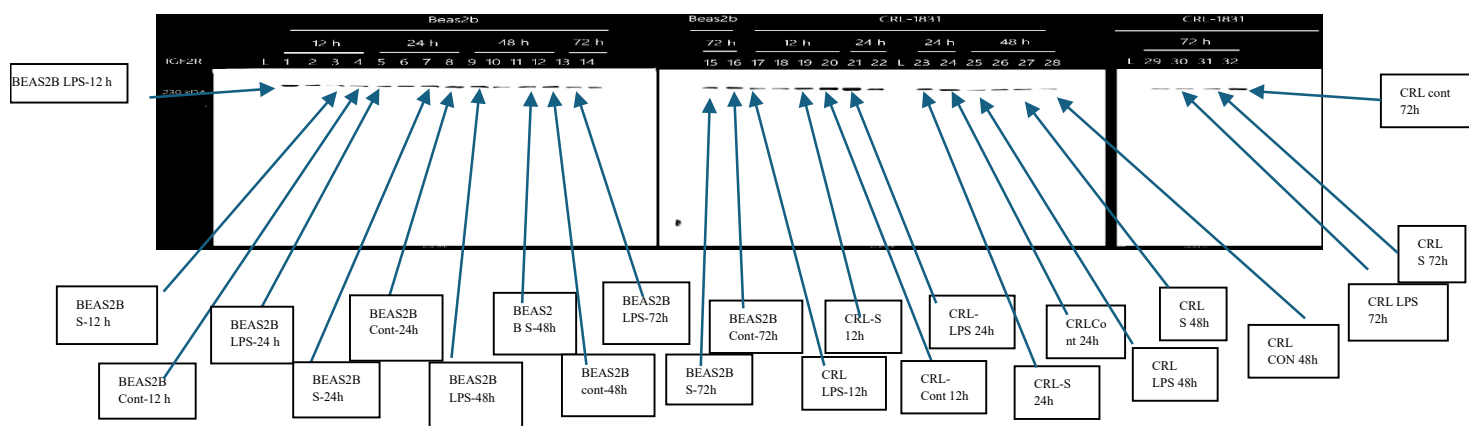

Supplement: Supplementary file 1 [file pathogens-15-00593-s001.zip › pathogens-4303116-supplementary.pdf]
